# Inhibition of Peptidyl Arginine Deiminase-4 Prevents Renal Ischemia-Reperfusion-Induced Remote Lung Injury

**DOI:** 10.1155/2020/1724206

**Published:** 2020-12-29

**Authors:** Mingjun Du, Lei Yang, Jianmin Gu, Jiawei Wu, Yiwen Ma, Tao Wang

**Affiliations:** ^1^Department of Thoracic Surgery, The First Affiliated Hospital of Nanjing Medical University, Nanjing 210029, China; ^2^Department of Cardiovascular Surgery, Renji Hospital, School of Medicine, Shanghai Jiao Tong University, 160 Pu-Jian Road, Shanghai 200127, China; ^3^Department of Intensive Care Unit, Chest Hospital, School of Medicine, Shanghai Jiao Tong University, Shanghai 200030, China; ^4^Department of Anaesthesiology, Shanghai Ninth People's Hospital, School of Medicine, Shanghai Jiao Tong University, Shanghai 200011, China; ^5^Department of Plastic and Reconstructive Surgery, Shanghai Ninth People's Hospital, School of Medicine, Shanghai Jiao Tong University, Shanghai 200011, China

## Abstract

Ischemia reperfusion (IR) can lead to acute kidney injury and can be complicated by acute lung injury, which is one of the leading causes of acute kidney injury-related death. Peptidyl arginine deiminase-4 (PAD4) is a member of the PAD enzyme family and plays a critical role in inflammatory reactions and neutrophil extracellular trap formation in a variety of pathological conditions. It has been reported that PAD4 inhibition can protect certain organs from ischemic injury. In this study, we aimed to understand the mode of action of PAD4 in renal ischemia-reperfusion-mediated acute lung injury. Bilateral renal pedicle occlusion was induced for 30 min followed by reperfusion for 24 h. A specific inhibitor of PAD4, GSK484, was delivered via intraperitoneal injection to alter the PAD4 activity. The pulmonary PAD4 expression, pulmonary impairment, neutrophil infiltration, Cit-H3 expression, neutrophil extracellular trap formation, inflammatory cytokine secretion, and pulmonary apoptosis were analyzed. We found that renal ischemia reperfusion was associated with pulmonary pathological changes and increases in neutrophil infiltration, neutrophil extracellular trap formation, and inflammatory cytokine secretion in the lungs of the recipient animals. Suppression of PAD4 by GSK484 reduced remote lung injury by mitigating neutrophil infiltration, neutrophil extracellular trap formation, apoptosis, and inflammatory factor secretion. Our findings demonstrate that specific PAD4 inhibition by GSK484 may be an effective strategy to attenuate distant lung injury complicating renal ischemia-reperfusion injury.

## 1. Introduction

Acute kidney injury is a common and severe complication of ischemia reperfusion (IR) in hospitalized patients and is associated with high mortality and morbidity [1–3]. Increasing evidence in patients indicates that acute kidney injury is a systemic disease characterized by systemic inflammatory responses and is frequently complicated by dysfunction of extrarenal organs, including the liver, heart, and lung [4, 5].

Among the types of distant organ failure that occur after acute kidney injury, acute lung injury (ALI) is the most common complication, which leads to a higher mortality rate than acute kidney injury alone [6, 7]. In recent decades, treatment of acute kidney injury-induced ALI has not been successfully improved; thus, a better understanding of the pathological mechanism of acute kidney injury-induced ALI would be helpful in developing a better therapeutic strategy and therefore improving the mortality rate. Inflammatory factors have been reported to be important effectors of acute kidney injury-induced remote organ crosstalk. Previous reports have demonstrated that renal IR injury increases the release of inflammatory molecules from the injured renal tissue into the systemic and pulmonary circulation, resulting in increased circulating levels of proinflammatory cytokines (e.g., IL-1b, IL-6, and TNF-a), and that these cytokines directly cause lung tissue edema and pathological damage [8–10]. It has been revealed that neutrophil infiltration is induced by inflammatory factors in the pathogenesis of distant lung injury [10, 11]. During the inflammatory process, infiltrating neutrophils release nuclear DNA and proteins known as neutrophil extracellular traps (NETs). The process of NET formation is called NETosis. NETs have been shown to be involved in various infectious diseases. NETs are web-like chromatin-based structures that are released into the extracellular environment and can act as pathogen traps [12]. However, NETs have also been implicated in excessive inflammation of noninfectious clinical conditions with resultant tissue damage [13]. Recently, it was reported that NETosis is a crucial mechanism in ALI pathogenesis [14]. Therefore, inactivation of infiltrating neutrophils and inhibition of NET formation might be efficacious strategies for the treatment of ALI.

Peptidyl arginine deiminases (PADs) are enzymes that accelerate the hydrolysis of peptidyl arginine to form peptidyl citrulline on histones and other biologically relevant proteins [15]. Peptidylarginine deiminase-4 (PAD4), a PAD isoform, is located in both the nucleus and cytoplasm and is highly expressed in neutrophils. PAD4 is regarded as a major regulator of NET formation via citrullination of histone H3 (CitH3), which leads to chromatin decondensation and release [3]. Furthermore, PAD4-mediated peptidyl citrullination is increased in several autoimmune and ischemic diseases, such as rheumatoid arthritis, multiple sclerosis, and myocardial infarction [16, 17]. Recent reports indicate that PAD4 activation contributes to acute tissue damage by directly exacerbating the inflammatory response [18].

GSK484 hydrochloride ([(3S,4R)-3-amino-4-hydroxy-1-piperidinyl] [2-[1-(cyclopropylmethyl)-1H-indol-2-yl]-7-methoxy-1-methyl-1Hbenzimidazol-5-yl]-methanone), a selective PAD4 inhibitor, has been used as an anti-ischemic compound and exerts inhibitory activity against PAD4 [16, 19]. We hypothesize that GSK484 could serve as an anti-ischemic reagent by inhibiting PAD4 and downstream responses of tissues to IR.

In this study, we examined inflammatory reactions and NET production induced by renal IR, the association between these responses and ALI risk, and whether selective inhibition of PAD4 attenuates inflammatory reactions and protects the lung tissue against acute kidney injury.

## 2. Materials and Methods

### 2.1. Reagents

[(3S,4R)-3-amino-4-hydroxy-1-piperidinyl][2-[1-(cyclopropylmethyl)-1H-indol-2-yl]-7-methoxy-1-methyl-1H-benzimidazol-5-yl]-methanone (GSK484) was obtained from Cayman Chemical (Michigan, USA). Antibodies against PAD4, Ly6g, CitH3, Neutrophil Elastase (NE), bcl-2, and caspase-3 were purchased from Abcam (Cambridge, UK). An antibody against GAPDH and rabbit IgG was acquired from Cell Signaling Technology (Beverly, USA). Interleukin 1*β*, interleukin 6, and tumor necrosis factor *α* (TNF-*α*) ELISA kits were provided by Thermo Fisher Scientific (Waltham, USA). A terminal deoxynucleotidyl transferase-mediated dUTP-biotin nick end labeling (TUNEL) assay kit was purchased from Roche Life Sciences (Indianapolis, USA). A blood urea nitrogen (BUN) was obtained from Thermo Fisher Scientific (Waltham, MA). A creatinine detection kit was obtained from Nanjing Jian Cheng Scientific (Nanjing, China).

### 2.2. Animals

Eight- to 10-week-old male C57BL/6 mice (weighing 20 to 25 g) were obtained from Shanghai Laboratory Animal Center, Chinese Academy of Sciences (SLACCAS, China). All experiments were conducted in accordance with the Guide for the Care and Use of Laboratory Animals published by the National Health and Family Planning Commission of the People's Republic of China. The animal protocol was approved by the Ethics Committee of the Shanghai Ninth People's Hospital, School of Medicine, Shanghai Jiao Tong University (Shanghai, China).

### 2.3. Construction of a Mouse Model of Renal IR Injury

Mice were anaesthetized by mask inhalation of 1.5% isoflurane and placed in the supine position. The fur was removed from the abdomen with depilatory cream. A midline incision was made, and the kidneys were exposed. Subsequently, the renal artery and vein were separated. The artery and vein were clamped for 30 minutes with an artery clamp, and then the clamp was removed to allow reperfusion for 24 hours. The incision was then closed, and the wound was cleaned and disinfected. Sham-operated mice underwent the same procedure without clamping of the artery and vein.

### 2.4. Experimental Protocols

C57BL/6 mice were randomly divided into four groups: the sham+vehicle group, sham+GSK484 group, IR + vehicle group, and IR + GSK484 group. GSK484 (4 mg/kg) was administered daily by intraperitoneal injection for 3 days before the operation. GSK484 was initially dissolved in 99.9% ethanol at a concentration of 25 mg/mL to generate a stock solution and then diluted 1 : 50 in sterile saline before injection (200 *μ*L/mouse) [16, 20]. The vehicle was prepared in the same way as GSK484 solution except GSK484 was omitted. The dose of GSK484 chosen based on previous studies.

### 2.5. Immunohistochemistry

Twenty-four hours after renal IR or sham operation, the lungs of the mice were collected and fixed in 4% formaldehyde, embedded in paraffin, and sectioned at a thickness of 5 *μ*m. The sections were exposed to 3% hydrogen peroxide for 10 min to suppress the endogenous peroxidase activity and then blocked with 3% bovine serum albumin for 30 min. The tissue sections were incubated with a rabbit anti-mouse PAD4 or anti-ly6g antibody (1 : 200 dilution) overnight at 4°C. The sections were then washed and incubated with an anti-rabbit IgG secondary antibody for 30 min at room temperature. The sections were counterstained with hematoxylin and developed using a diaminobenzidine (DAB) kit. The results were examined under a DP70 Microscope (Olympus, Inc., Tokyo, Japan).

### 2.6. Measurement of Renal Injury after IR Injury

Twenty-four hours after renal I/R injury, plasma blood urea nitrogen (BUN) and creatinine levels were measured using an enzymatic creatinine reagent kit according to the manufacturer's protocol. The intensity of the color was detected by a microtiter plate reader at wavelengths of 480 nm (BUN) and 510 nm (creatinine).

### 2.7. RNA Preparation and Real-Time Quantitative PCR (qRT-PCR)

Total RNA was extracted from lung tissues using TRIzol reagent (Takara, Japan) according to the manufacturer's instructions. Total RNA (1 𝜇g) was reverse transcribed into cDNA using a reverse transcription system following the manufacturer's protocol. The following primers were custom ordered from Sangon Biotech (Shanghai, China): PAD4: sense: 5′-GAGCAAGGATGGCCCAAGG-3′, antisense: 5′-GACAGTTCCACCCCAGTGAT-3′. GAPDH: sense: 5′-AGGTCGGTGTGAACGGATTTG-3′, antisense: 5′-TGTAGACCATGTAGTTGAGGTCA-3′. The gene expression was then determined using the 2-*ΔΔ*Ct method.

### 2.8. Histological Evaluation

Tissue sections were prepared as described above and stained with hematoxylin and eosin (H&E), and then light microscopy was used to examine the sections. Lung injury was objectively quantified as previously described [21]. In brief, alveolar congestion, hemorrhage, and infiltration of neutrophils in the air spaces or vessel walls and the thickness of alveolar wall/hyaline membrane formation were histologically analyzed. Lung injury was graded on a scale from 0 (minimal) to 4 (maximal), and the total score was calculated by adding the scores of each of these categories.

### 2.9. Wet/Dry (W/D) Weight Ratio

Fresh lung was weighed to obtain the wet weight. An oven was used to dry the lung tissues for 24 h at 80°C, and the tissues were weighed again to obtain the dry weight. The W/D weight ratio was used to evaluate pulmonary edema.

### 2.10. Protein Concentration in the Bronchoalveolar Lavage Fluid (BALF)

The trachea of the mice was dissected and cannulated with a 20 G catheter. The lungs were lavaged three times with ice-cold PBS (1.5 mL). BALF was collected and centrifuged at 3000 rpm for 10 min at 4°C, and total protein concentration in the supernatant was assessed with a QuantiPro™ BCA Assay Kit (Sigma-Aldrich, Missouri, USA).

### 2.11. ELISA

Twenty-four hours after renal IR or sham operation, the lungs of the mice were harvested, rinsed thoroughly with PBS, and homogenized. The homogenates were centrifuged at 400 × g for 30 min, and the supernatants were collected and stored at −80°C. Blood samples were collected from the carotid artery and incubated at room temperature for 30 min. Then, the samples were centrifuged at 400 × g for 30 min to harvest the serum. ELISA kits were used to measure BUN, creatinine, IL-1*β*, IL-6, and TNF-*α* production. The optical density was evaluated at 450 nm by using a microplate reader (BioTeK, Vermont, USA). BALF was prepared as described above, and the concentration of DNA bound to myeloperoxidase (MPO), a constituent of NET, was measured in BALF. Briefly, an antibody bound to the 96-well flat-bottom plate captured the MPO (Invitrogen, CA, USA), and the amount of DNA bound to MPO was quantified using a microplate reader (BioTeK, Vermont, USA).

### 2.12. Immunofluorescence

Tissue sections were prepared as previous study described [22]. Then, the sections were incubated with a rabbit anti-citH3 antibody (1 : 200 dilution) or a rabbit anti-citH3 antibody (1 : 200 dilution) and a Rabbit polyclonal to Neutrophil Elastase (NE, 1 : 200 dilution) overnight. After washing, the slides were incubated with fluorochrome-conjugated secondary antibodies at room temperature in the dark for 1 h. DNA was stained with 4′,6-diamidino-2-phenylindole (DAPI) for 1 min. Images were acquired using fluorescence microscopy (Zeiss, Germany).

### 2.13. Western Blot

Mouse lungs were homogenized in RIPA lysis buffer (Sigma-Aldrich) containing protease and phosphatase inhibitor mixtures (Roche Life Science). After estimating the protein concentrations with a bicinchoninic acid protein assay kit, an equal volume (40 *μ*g) of each sample was loaded onto SDS-polyacrylamide gels and electrotransferred onto PVDF membranes. The membranes were then blocked in 5% nonfat milk for 1 h and incubated overnight at 4°C with primary antibodies against bcl-2, cleaved caspase-3, or GAPDH. After washing with TBST, the membranes were incubated with HRP-conjugated secondary antibodies. Finally, the bands were detected using standard ECL reagent (Millipore, USA) and quantified by using ImageJ software version 1.8.0 (National Institutes of Health, USA).

### 2.14. Terminal Deoxynucleotidyl Transferase dUTP Nick-End Labeling (TUNEL) Assay

Twenty-four hours after renal IR or sham operation, lung tissue sections were prepared as described above. Cell death was detected by the in situ TUNEL assay according to the manufacturer's instructions. TUNEL-positive nuclei were observed with green fluorescein isothiocyanate fluorescence, and all cells presenting blue nuclear DAPI staining within six randomly chosen visual fields at high magnification were counted.

### 2.15. Statistical Analysis

The data are expressed as the means ± standard errors of the mean (SEMs). All statistical analyses were performed using Prism software (GraphPad Software Inc.). The data were analyzed by Student's *t*-test when the means of two groups were compared. One-way analysis of variance followed by Tukey's post hoc test was used for multiple comparisons. Each experiment was repeated at least three times, and *p* values <0.05 were considered to indicate statistical significance.

## 3. Results and Discussion

### 3.1. Results

#### 3.1.1. The PAD4 Expression in and Histological Damage to the Lungs after Renal IR Injury

**Figure 1:**
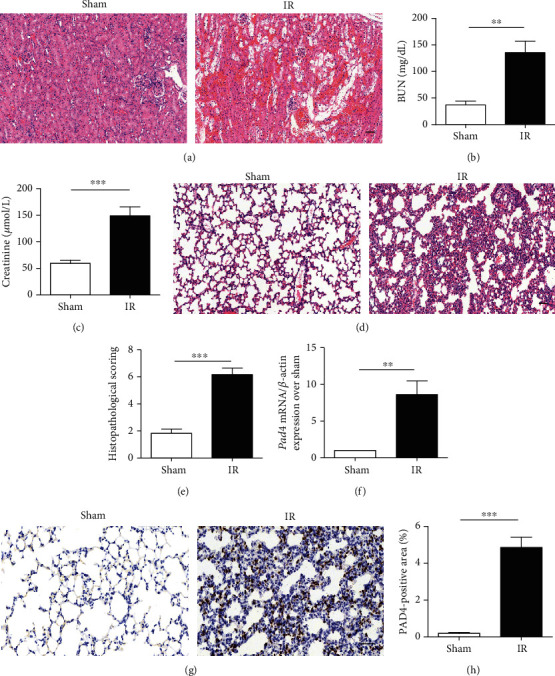
Kidney and pulmonary histopathological changes and lung PAD4 activity in renal ischemia-reperfusion- (IR-) treated mice. (a) Representative photographs of H&E-stained kidney samples from 24 h after renal IR injury (scale bar: 50 *μ*m). (b, c) Determination of blood urea nitrogen (BUN) and creatinine levels. (d) Representative photographs of H&E-stained lung sections from 24 h after renal IR injury (scale bar: 50 *μ*m). (e) Histological analysis of lung sections. (f) The PAD4 mRNA expression measured by quantitative RT-PCR. (g) The PAD4 expression evaluated by immunohistochemistry (scale bar: 50 *μ*m). (h) Analysis of the PAD4 expression. The values are the means ± SEMs; *n* = 6; ^∗∗^*p* < 0.01 and ^∗∗∗^*p* < 0.001.

Renal IR was induced by bilateral renal pedicle occlusion for 30 min followed by reperfusion for 24 h. After reperfusion, we first determined whether renal IR induced multitissue damage. Histological examination of kidney sections indicated greater tubular damage in the kidneys of IR mice than in those of sham mice (Figure 1(a)). In addition, serum creatinine and BUN levels were increased in the ischemic acute kidney injury group compared to the sham group (Figures 1(b) and 1(c)). To ascertain whether ischemic renal injury promoted the development of ALI, the lungs of recipient mice were histologically analyzed 24 hours after renal IR injury. As shown in Figure 1(d), renal IR injury resulted in severe pulmonary impairment, as evidenced by alveolar wall thickening, lymphocyte and neutrophil infiltration, pulmonary interstitial edema, and pulmonary hyaline membrane formation. Quantification of lung injury scores according to previously described criteria [21] revealed that lung injury was significantly higher in the IR group than in the sham group (*p* < 0.001, Figure 1(e)). We further measured the PAD4 expression in the lung tissue in response to renal IR injury, and the results showed an increase in PAD4 mRNA in the lungs of recipient mice compared to those of sham-operated mice (~8-fold, Figure 1(f)). Consistent with the increased transcription of PAD4, immunohistochemical analysis revealed the increased PAD4 protein expression in the lungs of mice subjected to renal IR compared to those of sham-operated mice. In contrast, the PAD4 expression was barely detectable in the absence of IR injury (*p* < 0.001, Figures 1(g) and 1(h)).

#### 3.1.2. PAD4 Inhibition Alleviated Renal IR-Induced Lung Tissue Injury

**Figure 2:**
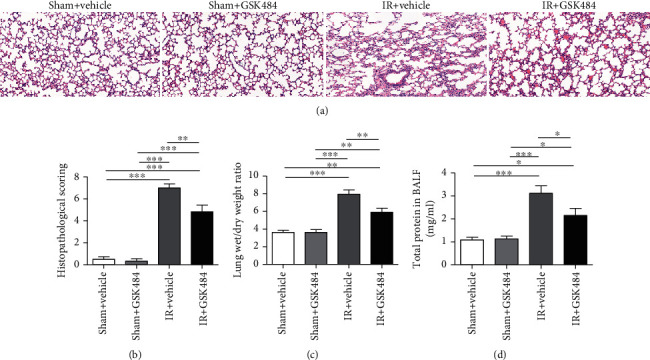
Effect of GSK484 on renal IR-induced pulmonary injury. (a) H&E staining of lung sections from different groups (scale bar: 50 *μ*m). (b) Histological analysis of different conditions. (c) W/D weight ratio of pulmonary tissue. (d) Protein leakage in the bronchoalveolar lavage fluid (BALF). The values are means ± SEMs; *n* = 6; ^∗^*p* < 0.05, ^∗∗^*p* < 0.01, and ^∗∗∗^*p* < 0.001.

We then examined the effect of GSK484 treatment. We evaluated alveolar congestion, hemorrhage, neutrophil infiltration, and alveolar wall thickness by histology. As indicated in Figure 2, the histopathological score of the IR + vehicle group was significantly higher than that of any other group (*p* < 0.001 compared with the sham + vehicle and sham + GSK484 groups and *p* < 0.01 compared with the IR + GSK484 group). These results indicate that the administration of GSK484 evidently mitigated the pathological alterations caused by renal IR insult. The pulmonary W/D weight ratio was markedly increased in renal IR-challenged lungs compared to the lungs of mice in the sham + vehicle and sham+GSK484 groups (Figure 2(c)*p* < 0.001). Total BALF protein levels in renal IR-treated animals were also significantly increased compared with those in sham + vehicle and sham+GSK484 animals (Figure 2(d)*p* < 0.001). However, GSK484 preconditioning significantly alleviated the renal IR-mediated enhancement of the pulmonary W/D weight ratio (Figure 2(c)*p* < 0.01 compared with that in the IR + vehicle group) and BALF total protein (Figure 2(d)*p* < 0.05 compared with that in the IR + vehicle group).

#### 3.1.3. GSK484 Treatment Inhibited Renal IR-Induced PAD4 and citH3 Expression in the Lungs

**Figure 3:**
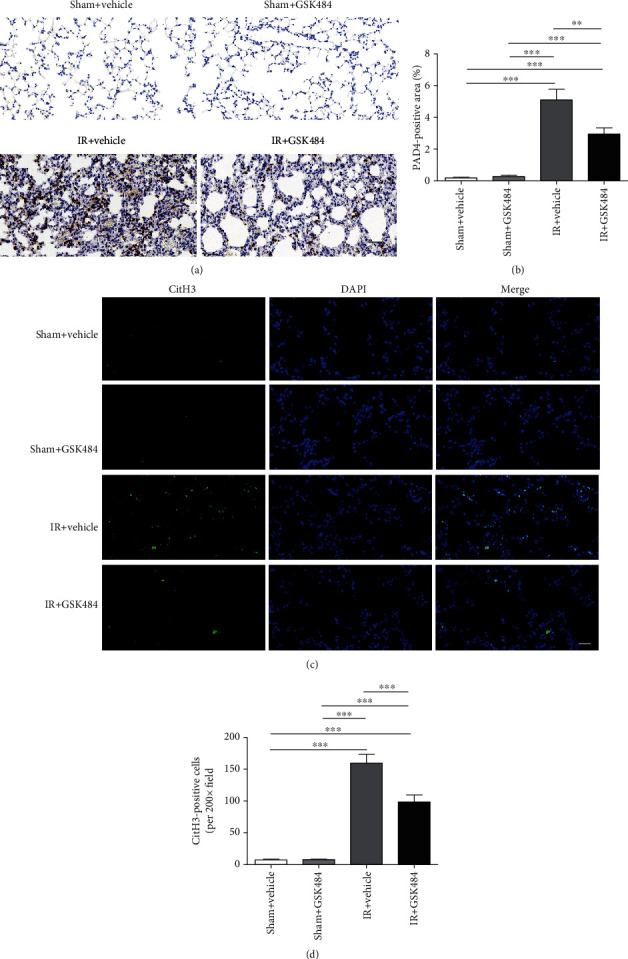
Effect of GSK484 on the PAD4 expression and citH3 accumulation in the lung after renal IR. (a, b) Representative images of immunohistochemical staining and analysis of PAD4-positive cells in lung sections from different groups (scale bar: 50 *μ*m). (c, d) Immunofluorescence images and analysis of H3cit-positive cells in the lung (scale bar: 20 *μ*m). The data are the means ± SEMs; *n* = 6; ^∗∗^*p* < 0.01 and ^∗∗∗^*p* < 0.001.

In our study, we used the specific PAD4 inhibitor GSK484 to inhibit PAD4 induction. The results showed that the GSK484 administration significantly mitigated renal IR-induced PAD4 production (*p* < 0.01 compared with that in the IR + vehicle groups; Figures 3(a) and 3(b)). Considering that PAD4 mediates citrullination of histone H3, we then performed immunofluorescence staining to test whether renal IR increases the citH3 expression in the lung. The results indicated that CitH3 was significantly upregulated after renal IR insult (*p* < 0.001 in comparison with that in the sham + vehicle and sham + GSK484 groups; Figures 3(c) and 3(d)). CitH3 levels were significantly decreased in the IR + GSK484 group compared to the IR + vehicle group (*p* < 0.001; Figure 3(d)).

#### 3.1.4. GSK484 Decreased NET Formation in the Lungs

**Figure 4:**
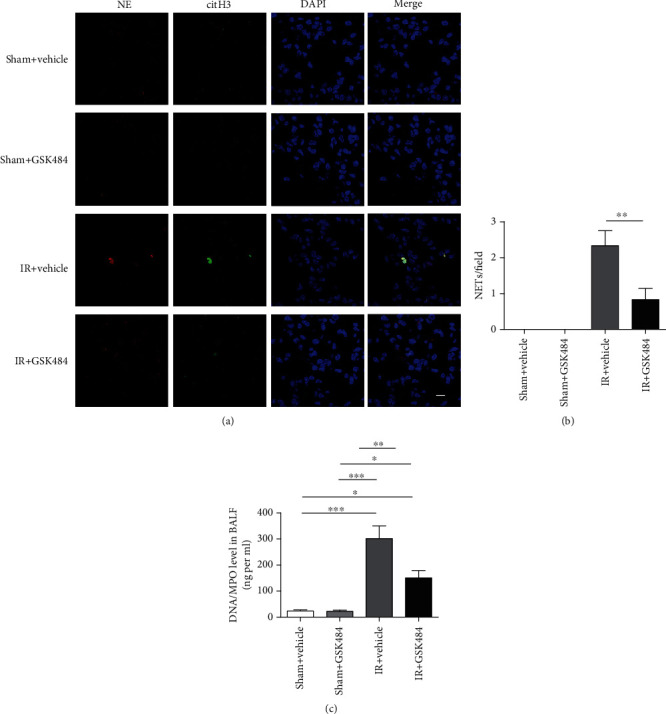
Neutrophil extracellular traps (NETs) in the mouse lung following renal IR-induced pulmonary injury. (a) Representative confocal images of NETs demonstrated colocalization of NE and CitH3 with diffuse DAPI staining nuclei. NETs: neutrophil extracellular traps; CitH3: citrullinated histone H3 (scale bar: 10 *μ*m). (b) Quantification of NETs in pulmonary sections from different groups. (c) Quantification of DNA/MPO levels in bronchoalveolar lavage fluid (BALF). The data are the means ± SEMs; *n* = 6; ^∗^*p* < 0.05, ^∗∗^*p* < 0.01, and ^∗∗∗^*p* < 0.001.

NETs are known to mediate various forms of tissue injury. To determine whether our renal IR-induced injury protocol also successfully induced NETs in the lungs, we examined the lung tissue by confocal microscopy to test for colocalization of citH3 and neutrophil granule proteins.  Figure 4(a) shows representative images of immunofluorescence staining for NETs in the lungs. Quantification of NET formation in different groups showed that NET formation seldomly occurred in the lungs of sham-operated mice, while increased colocalization of citH3 and NE (signifying the presence of NETs) was observed in the lungs of mice 24 h after renal IR injury. GSK484 treatment attenuated NET formation (*p* < 0.01 compared with that in the IR + vehicle group; Figure 4(b)). Next, we evaluated the DNA/MPO complex level in BALF. The results showed that renal IR induced an obvious NETosis in mouse lungs, as demonstrated by increased expression levels of DNA/MPO complex (*p* < 0.001 compared with the sham + vehicle and sham + GSK484 groups). In the presence of GSK484, the DNA/MPO level decreased dramatically compared with the IR + vehicle group (*p* < 0.01; Figure 4(c)). These results suggest that NET formation is strongly increased in the lungs during renal IR injury, and that specific inhibition of PAD4 can effectively repress NET formation.

#### 3.1.5. GSK484 Reduced Neutrophil Infiltration and Inflammatory Cytokine Secretion

**Figure 5:**
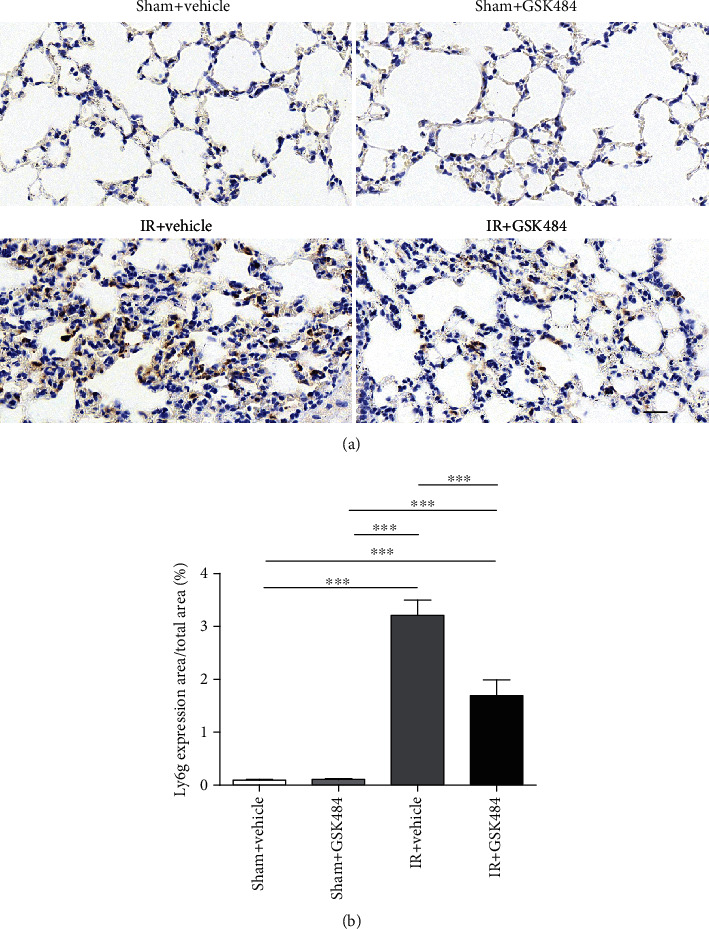
Effect of GSK484 on pulmonary neutrophil infiltration after renal IR-induced pulmonary injury. (a) Immunohistochemical staining for LY6G was performed on lung sections from different treatment groups (scale bar: 50 *μ*m). (b) Quantification of LY6G-positive cells. The values are presented as the means ± SEMs; *n* = 6; ^∗∗∗^*p* < 0.001.

**Figure 6:**
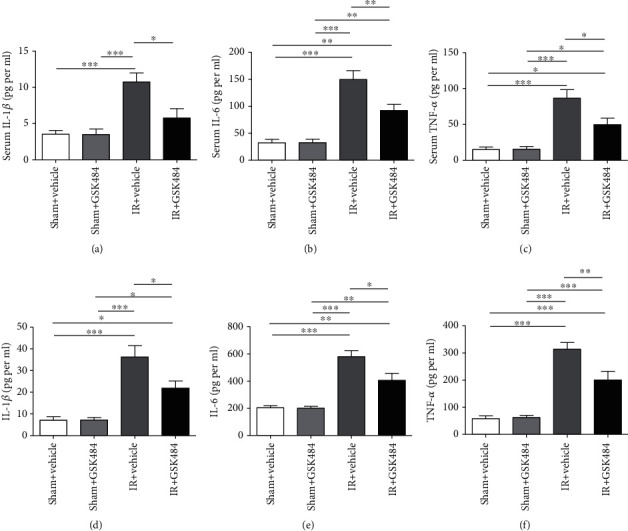
The influence of GSK484 on inflammatory cytokine secretion after renal IR injury. (a)–(c) Expression levels of IL-1*β*, IL-6, and TNF-*α* in the blood serum and (d)–(f) lung tissue homogenates. The values are presented as the means ± SEMs; *n* = 6; ^∗^*p* < 0.05, ^∗∗^*p* < 0.01, and ^∗∗∗^*p* < 0.001.

Ly6G is expressed predominantly on neutrophils, and the level of the Ly6G expression directly correlates with neutrophil infiltration. Therefore, we examined the levels of Ly6G, which indicates neutrophil infiltration in the lungs by staining. As shown in Figure 5, ly6g-positive neutrophils were barely detectable in the lungs of the sham + vehicle and sham + GSK484 mice. However, in the lungs of IR mice that received vehicle treatment, there was a large number of ly6g-positive cells. In contrast, GSK484 preconditioning markedly mitigated renal IR-induced enhancement of neutrophil infiltration in the lungs (Figures 5(a) and 5(b)). Plasma and lung tissue levels of IL-1*β*, IL-6, and TNF-*α* were measured 24 h after reperfusion to examine how GSK484 affected the systemic inflammatory response after renal IR. Renal IR markedly increased the level of each cytokine in both plasma and pulmonary tissue (*p* < 0.001 compared with that in the sham + vehicle and sham + GSK484 groups; Figures 6(a)–6(f)). The levels of IL-1*β*, IL-6, and TNF-*α* were dramatically decreased in the IR + GSK484 group compared with the IR + vehicle group (*p* < 0.01 or *p* < 0.001; Figures 6(a)–6(f)). Taken together, these results suggest that PAD4 inhibition can protect the lungs from renal IR-induced neutrophil infiltration and inflammatory cytokine production.

#### 3.1.6. Inhibition of PAD4 Conferred Protection against Renal IR-Induced Apoptosis

**Figure 7:**
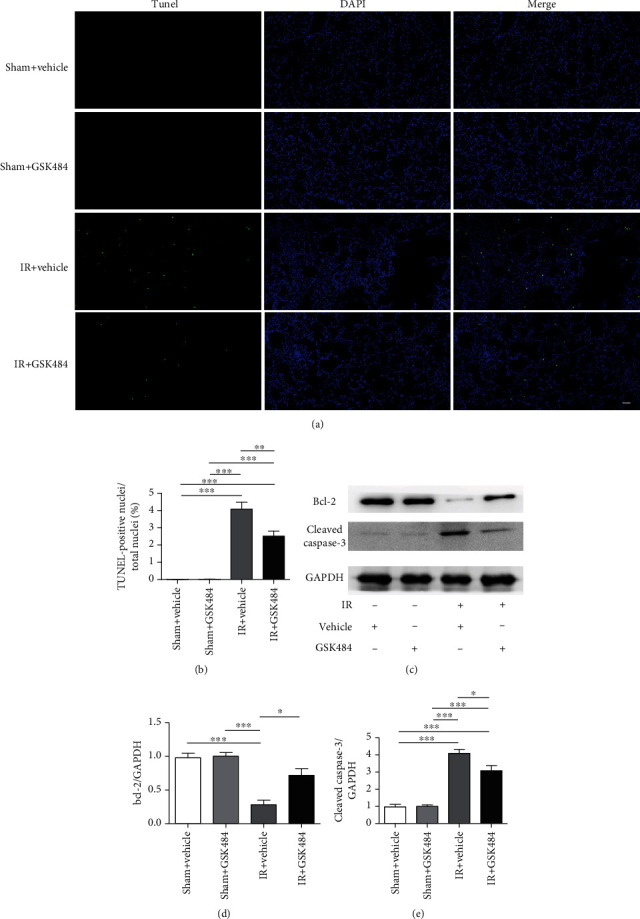
Effect of GSK484 on apoptosis in the lungs after renal IR-induced lung injury. (a) TUNEL staining was performed on lung sections (scale bar: 50 *μ*m). (b) Quantification of TUNEL-positive cells (*n* = 6). (c)–(e). Western blot analysis of the Bcl-2 and cleaved caspase-3 expression in the lungs (*n* = 3). The values are presented as the means ± SEMs. ^∗^p < 0.05, ^∗∗^*p* < 0.01, and ^∗∗^*p* < 0.001.

Acute kidney injury is known to exert a strong influence on apoptosis in lung tissues. TUNEL staining confirmed the occurrence of apoptosis in the affected lungs; the TUNEL staining intensity ranged from undetectable in sham-operated mice to well observed after renal IR injury (*p* < 0.001; Figures 7(a) and 7(b)). However, the number of apoptotic cells in lung tissues was dramatically decreased in the IR + GSK484 group compared with the IR + vehicle group (*p* < 0.01; Figures 7(a) and 7(b)). Furthermore, the Bcl-2 and cleaved caspase-3 expression in the different groups was measured by western blotting. We found that the protein expression of Bcl-2 was strongly decreased in the IR + vehicle group compared with the sham + vehicle and sham + GSK484 groups (*p* < 0.001; Figures 7(c) and 7(d)). Furthermore, GSK484 treatment significantly attenuated the IR-induced bcl-2 repression (*p* < 0.05 compared with that in the IR + vehicle group; Figures 7(c) and 7(d)). Moreover, the cleaved caspase-3 expression in the IR + vehicle group was increased significantly compared with that in the sham + vehicle group and the sham + GSK484 group (*p* < 0.001 Figures 7(c) and 7(e)). The IR + GSK484 group exhibited a decreased level of the cleaved caspase-3 expression compared with that in the IR + vehicle group (*p* < 0.05 Figures 7(c) and 7(e)). These results suggest that PAD4 inhibition can protect the lung from renal IR-induced apoptosis.

## 4. Discussion

In this study, we demonstrated that GSK484 preconditioning inhibited renal IR-mediated ALI by ameliorating the pulmonary inflammatory response. The critical results were as follows: (1) renal IR-induced pulmonary injury dramatically increased the PAD4 expression and activity in the lung, (2) direct PAD4 inhibition by GSK484 treatment markedly attenuated pulmonary histological damage and protected the lungs from inflammation (reduced neutrophil infiltration, attenuated CitH3 production and NET formation, and decreased serum and pulmonary inflammatory factor production), and (3) GSK484 significantly reduced apoptosis induced by renal IR in the lungs.

Renal IR-induced acute kidney injury contributes to the development of ALI. Recent studies have revealed several mechanisms of crosstalk between the kidneys and lungs, including water clearance dysfunction, increased pulmonary vascular permeability, neutrophil infiltration, and circulating and pulmonary IL-1, IL-6, and TNF-*α* expression [23–25]. Furthermore, extracellular histones have been suggested to play a crucial role in the development of pulmonary damage in acute kidney injury by promoting NET formation [26]. Our study confirmed increased neutrophil accumulation and enhanced NET formation in the lungs of mice with renal IR-induced pulmonary injury compared to those of sham mice. HE staining also confirmed that pulmonary damage was increased in the renal IR group compared to the sham group.

To date, five PAD subtypes have been identified in humans and rodents, and PAD enzymes can catalyze peptidyl-arginine to peptidyl-citrulline residues within a variety of protein targets. Therefore, PADs play an important role in a series of cellular signal transduction pathways [15]. Previous studies have indicated that PAD4 is primarily expressed in leukocytes and acts as an important regulator of NET formation [27]. Further investigations have revealed PAD4-mediated citrullination of certain proteins in several innate immunity and autoimmune diseases, such as colitis, lupus, rheumatoid arthritis, and multiple sclerosis [28, 29]. In recent years, PAD4 has attracted increasing attention due to its critical role in ischemic injury in various tissues, including the kidney and heart [16, 30, 31]. The PAD4 protein is barely expressed under normal conditions but can be activated in various tissues under pathological conditions [32, 33]. Our previous study demonstrated the destructive effects of PAD4 in myocardial infarction, as evidenced by enhanced neutrophil infiltration, activation of NET formation, aggravation of inflammation, and promotion of inflammatory cytokine secretion [16]. However, unlike the better characterized role of PAD4 in NET formation and the increase in PAD4 in autoimmune diseases and ischemic conditions, the role of PAD4 in the lung remains largely unclear. In the current study, we found that the PAD4 expression in the lung was strongly upregulated in response to renal IR injury. Given the relationships among PAD4, neutrophils, and NET formation, we hypothesized that PAD4 inhibition may protect the lungs from ischemic acute kidney injury-induced pulmonary dysfunction. Recently, many studies have used transgenic technology to investigate the function of PAD4 in various types of tissue damage [34, 35]. Nonetheless, gene transduction technology is still applicable in the clinic. Therefore, to evaluate the role of PAD4 in a simulated clinical setting, we decided to use the PAD4-specific inhibitor GSK484 to explore the role of PAD4 in renal IR-induced lung injury.

We found that the GSK484 administration significantly attenuated the overexpression of the PAD4 protein in the lung. In addition, we observed that GSK484 pretreatment dramatically attenuated the pulmonary damage induced by renal IR, as evidenced by reversal of hydrostatic alveolar edema, repression of increased capillary permeability, and prevention of pathological alterations. Furthermore, we observed less lung neutrophil infiltration in GSK484-treated mice than in control-treated mice upon renal IR. Considering that PAD4 regulates NET formation by converting peptidyl arginine residues to peptidyl citrulline on histone H3, we believe that the reduced lung CitH3 expression due to the repressed PAD4 activity might a surrogate marker of reduced NET formation in the lungs. Indeed, repression of the CitH3 activity was observed in the lungs of GSK484-treated mice due to PAD4 inhibition. Furthermore, immunofluorescence and ELISA confirmed the presence of fewer NETs in the lungs of GSK484-treated mice than those of vehicle-treated groups. These findings suggest that PAD4 is an effective pharmacological target for preventing pulmonary neutrophil infiltration and NET formation during renal IR insult.

During ALI, severe inflammatory reactions can enhance postrenal IR-induced pulmonary damage. Inflammatory molecules, such as IL-1*β*, IL-6, and TNF-*α*, have vital functions in the initiation and maintenance of lung inflammation [36]. Moreover, PAD4 has been shown to influence inflammation by modulating the cytokine expression in various tissues [16, 37]. Therefore, we further examined the secretion of several inflammatory cytokines in the lungs after renal IR injury. Our data indicated that renal IR treatment significantly enhanced the serum and lung tissue IL-1*β*, IL-6, and TNF-*α* production. Nevertheless, GSK484 attenuated renal IR-mediated proinflammatory cytokine secretion.

Increasing evidence has shown that severe inflammatory reactions promote pulmonary apoptosis following renal IR [36, 38]. Our current study revealed that PAD4 is involved in promoting proinflammatory cytokine secretion. Furthermore, 3 apoptotic markers were chosen to evaluate pulmonary apoptosis in our experiment, including TUNEL staining, cleaved caspase-3 expression, and bcl-2 expression. The results showed that pulmonary apoptosis was increased in the lungs after IR in the kidney and that inhibiting PAD4 significantly attenuated cell apoptosis in the presence of renal IR injury.

Our current study is not without limitations. For instance, further investigation of the exact mechanism by which PAD4 is activated after renal IR is required, and the mechanism underlying the effect of PAD4 and NETs in pulmonary injury was not explored in the present study and requires further investigation.

## 5. Conclusions

Our current study found that GSK484 has anti-inflammatory and antiapoptotic effects against ALI induced by renal IR injury and thereby demonstrated a novel protective mechanism against remote lung injury. These findings indicate that PAD4 inhibition may be a promising therapeutic avenue in acute kidney injury-induced remote organ crosstalk.

## Data Availability

The data used to support the findings of this study are included within the article.
